# Single center validation of routine blastocyst biopsy implementation

**DOI:** 10.1007/s10815-016-0792-3

**Published:** 2016-08-20

**Authors:** John B. Whitney, Mitchel C. Schiewe, Robert E. Anderson

**Affiliations:** 1 Ovation Fertility, Newport Beach, CA USA; 2 Southern California Center for Reproductive Medicine, Newport Beach, CA USA

**Keywords:** Blastocyst, Biopsy, PGS, Validation, Trophectoderm

## Abstract

**Purpose:**

The study aims to contrast the efficacy of trophectoderm biopsy preimplantation genetic screening (PGS)/vitrification (VTF)-all cycles to past treatment protocols. Specifically, do these applied technologies increase live birth rates on a per cycle/first transfer basis?

**Materials and methods:**

An observational, retrospective cohort study of first transfer outcomes was performed in two groups. Group 1 (PGS) included PGS/VTF-all cycles, and group 2 (no PGS) included the first transfer from non-PGS fresh cycles or VTF-ALL cycles. In group 1, all blastocysts were biopsied on days 5/6, vitrified and array CGH performed. Group 2 patients had embryo transfers on day 3 or day 5. All blastocysts were vitrified and warmed according to μS-VTF protocols. Clinical pregnancies and implantation were confirmed by ultrasound and live birth information attained. Results were stratified by age with donor cycles excluded, and to eliminate bias, the same groups were then validated on a per cycle basis. Chi-squared used to determine significance.

**Results:**

Analyzing 287 embryo transfers and 1,000+ PGS-tested blastocysts, an overall 97 % increase in live births favored group 1 (PGS). When utilizing PGS/VTF-ALL cycles, patients under 43 years old exhibited higher implantation, clinical pregnancy, and ongoing/live birth rates. Re-analyzing the data to include all cycles initiated revealed higher live birth rates in group 1 age groups ≤34 and 38–40 years old.

**Conclusion:**

Validating PGS on a per cycle basis eliminated data bias by including patients without blastocysts to biopsy or euploid embryos. Clearly, PGS uses blastocysts more efficiently to achieve success, while many women over 40 may benefit most by understanding why some failures occur.

**Support:**

None

**Electronic supplementary material:**

The online version of this article (doi:10.1007/s10815-016-0792-3) contains supplementary material, which is available to authorized users.

## Introduction

A healthy baby is the end goal for patients choosing assisted reproduction technologies (ART; [1]). The potential or percent chance for achieving a live birth often guides the patients’ decision process for treatment. Success is generally evaluated by clinical pregnancies, live births, and implantation efficiency. These outcomes are influenced by cycle initiation, ovarian stimulation, oocyte quality, embryo culture, and transfer selections [2], as well as patient age. Therefore, comparisons among different clinics are difficult to assess and potentially misleading [3]. Fundamentally, it is an individual clinics’ responsibility to institute a comprehensive quality management program for self-evaluating and verifying their efficacy of procedural success, especially when applying new technologies. Furthermore, it is particularly beneficial to report and evaluate a patient’s end goal in terms of singleton live births, the best determinant of treatment success [1].

To accurately assess pregnancy outcomes, one must understand the intricacy required to achieve a pregnancy via in vitro fertilization (IVF). Even when laboratory procedures are optimized, success is highly dependent on proper embryo selection for transfer [4]. Embryo selection is imperative for improved pregnancy rates, but is subject to variable technician and program-specific criteria [5]. Subjective morphology grading [4] has been a widely accepted and reliable standard to judge embryo quality. The international movement toward single embryo transfers (SET) has increased the pressure to improve embryo selection criteria [6]. Yet, our ability to more accurately choose embryos with a high probability for success has proven difficult utilizing morphologic assessments alone [7].

Our clinic experienced a fundamental shift in treatment ideology in 2012 when we moved from a standard fresh blastocyst transfer treatment regime (strictly utilizing the American Society for Reproductive Medicine (ASRM) guidelines) to the application of day 5/6 blastocyst biopsy with vitrification-all cycles. Preimplantation genetic screening (PGS) ploidy determination was followed by subsequent euploid vitrified-warmed embryo transfer emphasizing single embryo transfer. The rapid inclusion of blastocyst biopsy in ART, in conjunction with PGS and vitrification-all cycles in our clinic, prompted us to question if our overall success had improved. This change in treatment plan potentially created a selection bias, resulting in an increase in pregnancies per transfer by eliminating patients failing to produce a normal blastocyst. To fully validate the advantages of blastocyst biopsy with PGS, we sought to normalize and contrast pregnancy outcomes of non-PGS and PGS cycles over an 18-month duration before and after, respectively, our inclusion of established blastocyst biopsying procedures.

## Materials and methods

### Study design

Using an observational retrospective cohort analysis, we strived to evaluate the efficacy of our blastocyst biopsy program. Prior to July 2012, PGS cycles accounted for <20 % of all cycles initiated in our clinic. With the rapid inclusion of blastocyst biopsy beginning in January 2012, by July 2012, PGS aneuploidy screening accounted for over 90 % of all initiated cycles in our clinic. This study aimed to evaluate the shift from utilizing morphological quality grading only for fresh embryo transfer selection to morphology combined with genetic analysis for vitrified warmed embryo transfer cycles.

Two study arms were evaluated, the first was per transfer, and standard implantation analysis and live birth per transfer were analyzed. A transfer must have been attempted for inclusion in this analysis. Since many cycles fail to attain a euploid blastocyst for transfer, the traditional measures used to evaluate live birth per transfer are inherently biased. The second arm was per cycle, and all cycles that received stimulation were included. This validation approach aimed to eliminate fundamental inaccuracies when comparing PGS tested embryo transfer data to untested embryo transfer cycles.

In an effort to further reduce bias, only the patients’ first transfer attempt was used for comparisons. If a cycle whose intended transfer did not result in a transfer, the outcome was classified as not pregnant and included for statistical analysis in the per cycle arm. The study was IRB approved for retrospective analyses.

### Cohort determination

All cycles included in this study were initiated from January 2011 to December 2013. All cycles initiated from July 2012 through December 2013 were included in group 1 (PGS). Of all these cycles, only those that met the exclusion criteria were excluded. All patients enrolled in group 1 (PGS) initiated a cycle intending the use of PGS and transfer of only euploid blastocysts.

All cycles initiated from January 2011 through June 2012 were included in group 2 (no PGS), and all cycles meeting exclusion criteria were excluded. The decision to separate two cohorts during this time period was justified to eliminate a population bias, as all cycles after July 2012 were encouraged to utilize blastocyst biopsy with a significant reduction in fresh ET occurring. Population analysis was performed to identify differences between the two groups.

### Population parameters

#### Inclusion criteria

All cycles performed standard IVF stimulation protocols, established embryo culture practices, intracytoplasmic sperm injection (ICSI), day 5 or 6 biopsy (group 1), and microSecure vitrification [8, 9] of all fair to excellent quality blastocysts (i.e., ≥3BB) (group 1 and group 2—cryo-all). All cycles, including canceled cycles, were used in the per cycle analyses.

**Fig. 1:**
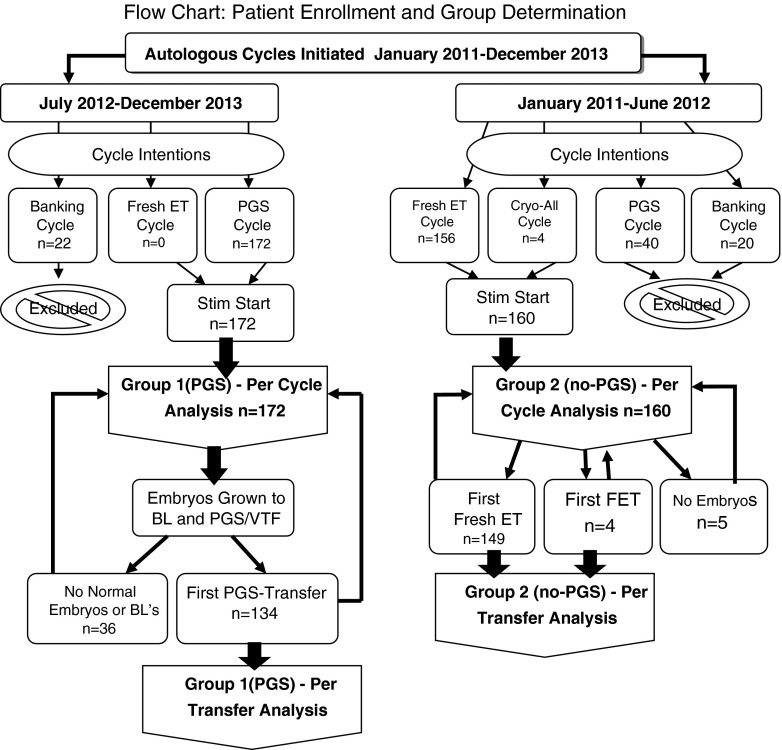
Experimental flow diagram indicating patient enrollment and treatment group determination assignments, revealing the total number of patients included or excluded from the study

Group 1 (PGS) and group 2 (no PGS) patients used autologous oocytes, performed standard IVF/ICSI cycles, and morphology dictated transfer selection for group 2 (no-PGS) and morphology with a euploid genetic result dictated group 1 (PGS) transfer selection, unless gender was requested. Inclusion criteria for the per cycle and per transfer analysis arms are displayed in a flow chart (Fig. 1). There were no cycles in group 1 (PGS) that chose to transfer untested embryos. Likewise, every cycle initiated after June 2012 grew all embryos to the blastocyst stage for biopsy. Every cycle included in group 1 (PGS) intended and continued for PGS regardless of any embryology or patient factors. If no blastocysts resulted, all embryos were discarded, and the cycle was classified as not pregnant.

Cycle intentions were made at the start of controlled ovarian hyperstimulation and any cycle which received medication, but was canceled for any reason, was included as a negative result in the per cycle arm. One cycle in group 2 was converted to an intrauterine insemination (IUI) and included in the per cycle analysis as a negative, as the IUI did not result in pregnancy. Every attempt was made to include cycles during the designated time periods, and every cycle which initiated for transfer was included.

#### Exclusion criteria

Both group 1 (PGS) and group 2 (no PGS) were designed to reflect a patients’ intention for transfer, thus no embryo or oocyte banking cycles were used for calculations in either group (Fig. 1). If a patient’s egg retrieval occurred in the designated time frames, but no resulting embryos were transferred due to spontaneous natural pregnancy following treatment (*n* = 3), their cycle was excluded from their respective group and termed banking. All single gene diagnosis cycles and/or oocyte cryopreservation cycles were excluded. All PGS cases that initiated during the group 2 (no PGS) time frame were excluded. There were no exclusions for any embryology, patient, or cycle parameters, with every cycle that initiated for transfer being included.

### Embryo culture, grading, biopsy, and PGS testing

Using MCO-5 M mini Sanyo/Panasonic tri-gas incubators (5 % 0_2_/5.3–6.0 % CO_2_), we group cultured up to five embryos per 25 μL droplet of Global™ medium (LG; Life Global, Guilford, CT) supplemented with 7.5 % synthetic protein supplement under Ovoil™ (Vitrolife, Englewood, CO) until blastocyst biopsy [10]. All oocytes retrieved were evaluated for maturity and had ICSI performed 2–6 h post-egg retrieval [9]. Embryos were initially evaluated on day 3; laser zona dissection was performed using a 1480-nm diode laser (Zilos-tk™; Hamilton Thorne, Beverly, MA), and embryo incubation continued until day 5/6 evaluations [10]. The zona opening created on day 3 allowed trophectoderm (TE) to prematurely rupture through a 10–12-μm furrow in the zona. The same laser was used on day 5/6 for biopsying using a combination of laser pulses and mechanical separation to achieve 3–10 TE cells [10] used for array CGH (Genesis Genetics, Plymouth, MI). Blastocysts were graded at biopsy using a modified Gardner scale [11]. The modification was to account for premature hatching: grade 3 = 5–10 % TE extrusion (full blastocyst), grade 4 = 10 %–50 % TE extrusion (expanded blastocyst), and grade 5 ≥ 50 % TE extrusion (hatching blastocyst). Inner cell mass (ICM) and TE were independently graded from top quality “A” to fair quality “B” and poor quality “C” with the first letter in the grade assigned to the ICM and the second to TE. A grade of 3BB or better was required to initiate biopsy, and all embryos were allowed to culture to day 6 for further expansion to meet biopsy criteria.

### Vitrification and embryo transfer

Fair to excellent quality blastocysts (≥3BB grade) were vitrified on day 5 or day 6 using microSecure-VTF in non-DMSO vitrification (VTF) solutions (Innovative Cryo Enterprises, Linden, NJ; 9). Aseptic microSecure VTF was performed using a 3-step dilution (5 min/5 min/1 min); individual blastocysts were loaded into 300 μm ID flexipettes (Cook Medical, Spencer, IL; 3 μl volume); flexipettes were then dried and inserted tip first into prelabeled 0.3 ml CBS™ embryo straws; the straw weld sealed and plunged directly into LN_2_ [8]. Rapid warming was achieved by direct placement of the vitrified flexipettes into a 37 °C 0.5 M sucrose bath [9]. Within 10 s, each blastocyst was pipette directly from the flexipette into an open 200 μl droplet of 1.0 M sucrose solution and then transferred into 100 μl droplets under oil for 3 min intervals. Embryos were serially diluted in declining sucrose solutions (T1–T4), before isotonic equilibration in Hepes-LG medium. Warmed blastocysts were then cultured in LG medium + protein for 1–3 h prior to vitrified ET (VFET).

All VFET cycles were hormone replacement cycles using oral estradiol, estradiol patches, or intramuscular (i.m.) estradiol valerate followed by i.m. progesterone in oil. Progesterone in oil was started when endometrial thickness was >8 mm after documentation of serum progesterone level of <1.0 ng/ml. VFET was performed after 5 days of intramuscular progesterone administration. All transvaginal ultrasound guidance ET procedures were performed by a single physician. Pregnancies were initially tested 10 days post-ET and implantation subsequently assessed by transvaginal ultrasound beginning 4 weeks later. Live births were confirmed by written or oral communication with patients.

### Statistical analysis

Initial comparisons for implantation, clinical pregnancies, live births, and spontaneous abortions were calculated per first transfer attempt. This analysis was the per transfer arm of the study and included only cycles that resulted in a transfer. To eliminate study bias, all comparisons between groups was performed on a per cycle initiation to outcome basis. This second analysis was the per cycle arm of the study and included all cycle stimulation starts. The per cycle analysis included the per transfer data, all canceled cycles, cycles that did not produce any embryos, and/or cycles where all embryos were aneuploid. This analysis was used to compare the groups with stimulation start as the denominator. Student’s *t* test was used to assess the significance of potential patient population differences between group 1 and group 2 traits (e.g., average age, stimulation protocol, average follicle count, and day 3 FSH). Alternatively, chi-squared analyses were performed to contrast differences in pregnancy outcomes (e.g., clinical pregnancy, implantation, and live birth rates).

## Results

**Fig. 2:**
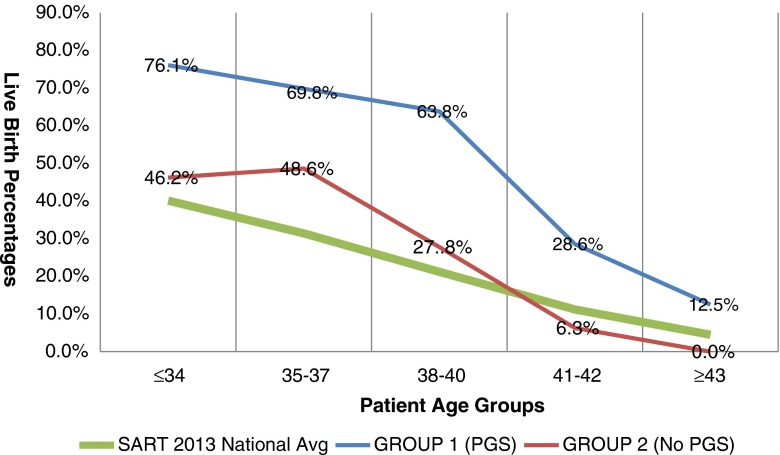
Live birth rates per cycle starts were compared between group 1 and group 2. The benefit of transferring ploidy screened embryos in group 1 is contrasted to both untested embryo transfers within a single clinic (group 2) and to the 2013 SART national live birth averages. Group 1 clearly displays an increase in live births which reinforces the benefits gained by the routine IVF commitment to the clinical application of PGS

Population comparisons were conducted between group 1 and group 2 to assess variations in patient parameters (Table 1). No differences in age, stimulation protocol, or antral follicle count were revealed; however, day 3 FSH levels were higher (*p* < 0.05) in group 2 (no PGS) women in the 35–37 and ≥43 age groups. Group 1 (PGS) achieved higher (*p* < 0.05) implantation and clinical pregnancy rates when sorting by age groups under 43 (Table 2). Live birth rates were statistically higher for age groups <34, 38–40, and 41–42-year-old women receiving single euploid ET. Re-analyzing the data to include all cycles regardless of outcome per first transfer showed significance for age groups ≤34 and 38–40 (Table 3). Utilizing the 2013 CDC/SART averages for live birth per cycle, we contrasted them to group 1 (PGS) and group 2 (no PGS) live birth outcomes (Fig. 2). An increase in average pregnancies was observed in each age group with a 97 % increase in average live birth rates favoring group 1 (PGS). Additionally, fewer (*p* < 0.01) embryos were transferred in group 1 (PGS) patients under 43 years old (Table 4), whom also had a lower overall spontaneous abortion (SAB) rate (*p* < 0.05; Table 5). Thus, the overall efficacy of pregnancy establishment/success was greater in group 1 (PGS).

**Table 1:** Population comparison of patients in Group 1 versus Group 2

	Mean age	% Antagon	% Agonist	Mean # follicle	Mean FSH
Age ≤ 34	NS 31.6 vs 31.5	NS 63 vs. 67 %	NS 37 vs. 33 %	NS 17.1 vs. 15.6	NS
Age 35–37	NS 36.0 vs. 36.0	NS 70 vs. 83 %	NS 30 vs. 17 %	NS 16.4 vs. 13.7	*p* < 0.05 6.1 vs. 7.6
Age 38–40	NS 39.0 vs. 39.1	NS 89 vs. 92 %	NS 11 vs. 8 %	NS 11.9 vs. 16.1	NS
Age 41–42	NS 41.1 vs. 41.6	NS 100 vs. 100 %	NS 0 vs. 0 %	NS 11.8 vs. 9.9	NS
Age ≥43	NS 43.6 vs. 43.4	NS 100 vs. 100 %	NS 100 vs. 100 %	NS 10.3 vs. 8.3	*p* < 0.05 6.4 vs. 9.1

Statistical significance determined by *t* test calculations; *NS* not significant *p* > 0.05

**Table 2:** Per transfer arm: transfer comparisons

	Group 1 (PGS)	Group 2 (no PGS)	
Age	Clinical Preg per ET		*p* value
≤34	88.4 % 38/43	51.6 % 33/64	*p = ≤0.01
35–37	85.4 % 35/41	62.5 % 20/32	*p = ≤0.05
38–40	83.8 % 31/37	37.1 % 13/35	*p = ≤0.01
41–42	66.7 % 8/12	6.7 % 1/15	*p = ≤0.01
43+	100.0 % 1/1	0.0 % 0/7	*p* = 0.11
Age	Live birth per ET		*p* value
≤34	81.4 % 35/43	46.9 % 30/64	*p = ≤0.01
35–37	73.1 % 30/41	53.1 % 17/32	*p* = 0.08
38–40	81.1 % 30/37	28.6 % 10/35	*p = ≤0.01
41–42	66.7 % 8/12	6.7 % 1/15	*p = ≤0.01
43+	100.0 % 1/1	0.0 % 0/7	*p* = 0.111
Age	Implantation		*p* value
≤34	84.6 % 44/52	39.5 % 49/124	*p = ≤0.01
35–37	78.6 % 44/56	36.6 % 26/71	*p = ≤0.01
38–40	81.4 % 35/43	23.6 % 17/72	*p = ≤0.01
41–42	72.2 % 13/18	2.6 % 1/38	*p = ≤0.01
43+	100.0 % 1/1	0.0 % 0/19	*p = ≤0.05

*Chi-square was used to determine differences between groups within rows

**Table 3:** Per cycle arm: the comparative effect of implementing blastocyst biopsy/PGS on a per cycle start basis

		Group 1 (PGS)			Group 2 (no PGS)				
Age	# cycles	# ET	# live birth	Live birth/cycle		# cycles	# ET	# live birth	*p* value
≤34	46	43	35	76.1 %	*46.2 %	65	64	30	*p = ≤0.01
35–37	43	41	30	69.8 %	48.6 %	35	32	17	*p* = 0.07
38–40	47	37	30	63.8 %	*27.8 %	36	35	10	*p = ≤0.01
41–42	28	12	8	28.6 %	6.3 %	16	15	1	*p* = 0.124
43+	8	1	1	12.5 %	0.0 %	8	7	0	*p* = 1.0

*Differences in live birth rate were determined by chi-squared analysis

**Table 4:** Average number embryos transferred

Age group	Group 1	Group 2	*p* value*
≤34	1.2	1.9	*p* < 0.01
35–37	1.4	2.0	*p* < 0.01
38–40	1.2	2.5	*p* < 0.01
41–42	1.2	2.5	*p* < 0.01
43+	1.0	2.7	*p* = 0.12

*t test used to determine significance between columns within row

**Table 5:** Overall spontaneous abortion rate comparisons between PGS (group 1) and non-PGS (group 2) treatments, independent of age

	Group 1	Group 2	*p* value
Average	4.4 %	12.9 %	*p* ≤ 0.05

## Discussion

This study aimed to determine the efficacy of a progressive treatment change utilizing blastocyst biopsy with PGS and perform a detailed, unbiased self-assessment. The results generated affirm the safety of blastocyst biopsy through reduced SAB results and the lack of abnormalities being reported among newborns. These findings confirm the belief that blastocysts are the ideal stage for biopsy intervention [12] and that PGS, in conjunction with vitrified warmed embryo transfers, is an ideal option to efficiently succeed in transferring a single embryo. The overall analysis of clinical pregnancy per first transfer was higher for women <43 years using the PGS/VTF treatment protocol (group 1). Most importantly, implantation for all age groups improved (*p* < 0.05) in group 1 (PGS), revealing better embryo utilization, strengthening our decision to move to elective single embryo transfers as our standard of care.

Ultimately, our study objective was to determine whether a blastocyst biopsy/vitrification program benefits all patients on a per cycle analysis basis. Our results not only showed an increase in pregnancies in all age groups when analyzing per cycle start, but it also effectively reduced miscarriage rates per transfer. In turn, our overall take home baby rate did increase 97 % per first transfer attempt. Not surprisingly, the per cycle analysis for the live birth rates was not different for patients over 4 years old. However, our failure to achieve significance at *p* < 0.05 for women in the 35–37 year age group was unfortunately traced back to a single group 1 (PGS) patients’ elective termination (i.e., abortion), not due to chromosomal abnormalities or pregnancy failure. Nonetheless, we feel the ability to diagnose potential cycle failures and drastically reduce the rate of pregnancy losses (i.e., SAB) strongly justifies the intervention needed to biopsy a blastocyst. Additionally, the ability to effectively control and reduce potential multiple births, to less than 2 %, in good prognosis patients through the routine usage of a single euploid embryo encourages the application of PGS to maintain a high standard of patient care. The transferring of euploid blastocysts within this study supports the importance of ploidy screening for increasing implantation, and ultimately, live birth success.

It is noteworthy to recognize that our comparisons were not run simultaneously as a randomized controlled PGS trial. Indeed, dramatically different time frames were evaluated as the clinic did not offer PGS to all patients in 2011, and by mid-2012, all cycles initiated were intended for and subsequently underwent PGS testing. The groups were evaluated for baseline differences, and no capital improvements or significant changes to patient care or laboratory protocols were implemented during the time frames in question other than blastocyst biopsy. Scott and coworkers have previously shown improvements implementing trophectoderm biopsy within a randomized controlled trial (RCT, [13]), complementing the results found in this analysis. Zhu and coworkers first reported the general advantages of vitrification-all cycles improving pregnancy outcomes [14]. Although we clearly found this to be true early on for PGS cycles compared to delayed fresh ET on day 6 (unpublished data), this study did not specifically compare PGS/FET to FET without PGS. However, the empirical data generated in our VTF validation analyses [9] did support the concept that our VFET cycles were equal to or better than fresh ET. Finally, some patient parameters (e.g., physical characteristics, stimulation factors) were not evaluated between groups, as many parameters were not collected for all cycles and ultimately not evaluated in this study.

Fundamentally, this study was a validation verification intended to eliminate the bias generated through accounting for and transferring only euploid embryos, thus allowing for a fair, standardized analysis of live births per cycle initiation. Our clinic’s goal to develop and optimize a path for single embryo transfer as a standard practice was in-line with an international movement toward decreasing multiple births associated with ART treatments. By comparing the transfer of euploid screened embryos to previously unscreened fresh or frozen transfer cycles, our normalized data clearly showed significant improvements in live birth outcomes.

This observational study was our best attempt to self-evaluate and validate a change in patient care. It is well understood that this was neither a prospective nor randomized study, but it has generated relevant data which supports other RCTs regarding the efficacy of blastocyst biopsy with aneuploidy screening. Our study also chose to only calculate per first transfer. A per cohort analysis could presumably achieve a higher live birth percentage by including all transfers for a single patient. However, we question, when calculating per cohort and utilizing ASRM guidelines for transfer, what increase in adverse outcomes (i.e., biochemical pregnancy, SAB, or multiple gestations) may patients endure. Undoubtedly, PGS adds cost to the cycle, but what costs can be placed on the emotional distress and trauma endured by women experiencing repeated failures and fetal loss. Thus, a true assessment of cost per live birth comparing PGS versus untested embryo transfer has not been performed. Overall, this study defends our current clinical practice, providing optimism that we are offering our patients a justified, quicker, more emotionally balanced path to pregnancy success. Factoring in the limited pregnancy loss(es) and increased singleton live births, we are now able to offer a more enjoyable, safer, and positive IVF experience for the majority of our patients.

## Electronic supplementary material

Below is the link to the electronic supplementary material.

ESM 1 (PDF 496 kb)
